# Supplementary material for: External cephalic version at 38 weeks’ gestation at a specialized German single center
Source: PLoS One. 2021 Aug 30;16(8):e0252702. doi: 10.1371/journal.pone.0252702 (PMC8405029; doi:10.1371/journal.pone.0252702)
Supplement: Attachment — Submitted filename: Response to Reviewers.docx [file pone.0252702.s001.docx]

**Rebuttal Letter PONE-D-20-24091**

Dear Editor, dear Reviewers,

Thank you for your helpful and supporting input and questions you raised in this review process. Addressing each point improved the manuscript. Hopefully, your demands were met within our revision process.

Below, you can find a point-to-point statement. (Answers in *italic*)

Yours sincerely,

Ann-Sophie Zielbauer

1. Please ensure that your manuscript meets PLOS ONE's style requirements, including those for file naming. The PLOS ONE style templates can be found at

*Journal requirements were double-checked.*

2. Thank you for stating the following in the Competing Interests section:

'I have read the journal's policy and the authors of this manuscript have the following competing interests:
F.L. is first vice president of the German Society for Gynaecology and Obstetrics (DGGG), council member of the European Board and College of Obstetrics and Gynaecology (EBCOG) and Executive Board Member und Committee chairman of the International Federation of Gynecology and Obstetrics (FIGO).
F.L. and L.J. worked on the first German S3-Guideline for cesarean section published in 2020.
A.Z. declares no relevant conflicts of interest.'

a. Please confirm that this does not alter your adherence to all PLOS ONE policies on sharing data and materials, by including the following statement: "This does not alter our adherence to PLOS ONE policies on sharing data and materials.” (as detailed online in our guide for authors [http://journals.plos.org/plosone/s/competing-interests](about:blank)).  If there are restrictions on sharing of data and/or materials, please state these.

Please note that we cannot proceed with consideration of your article until this information has been declared.

*F.L. is first vice president of the German Society for Gynaecology and Obstetrics (DGGG), council member of the European Board and College of Obstetrics and Gynaecology (EBCOG) and Executive Board Member und Committee chairman of the International Federation of Gynecology and Obstetrics (FIGO). F.L. and L.J. worked on the first German S3-Guideline for cesarean section published in 2020. A.Z. declares no relevant conflicts of interest. This does not alter our adherence to PLOS ONE policies on sharing data and materials.*

b. Please include your updated Competing Interests statement in your cover letter; we will change the online submission form on your behalf.

*We included the Update in our cover letter.*

3. Review Comments to the Author

Reviewer #1: title - too long

*Thank you for your profound comments. They were very helpful in improving our manuscript.*

*The title was shortened. The new title is: “External cephalic version in 38 weeks’ gestation in a specialized German single center”.*

keywords - too many

*Less important key words were deleted.*

introduction - ECV is mostly performed before 37 GA?

*The wording was infelicitous. Despite recommendations for performing ECV after 37 weeks of gestation, a lot of departments still offer ECV before 37 weeks in Germany. Citations were included in the revised manuscript.*

LINE 67 - provide reference for most ecv performed before 37

*Citations were included in the revised manuscript.*

line 68-70 - is redundant

*The passage was shortened, we focused on more present literature.*

ECV not offered line 79 - provide reference

*Citations were included in the revised manuscript. Kohls et al. carried out an anonymized online survey asking about preferred primary intervention for ECV in Germany in 2018. Although it seems that ECV is offered nationwide, only for 61% ECV is the preferred primary intervention, for 12.4% of the surveyed hospitals CS is still the preferred intervention.*

line 79-81 - more studies to be published??? numerous studies regarding predictors of success. the authors should replace outofdate reference (years 2000-2010) regarding success rate (ezra, ben meir) and replace with more updated reference (31312960, 30941816)

*Outdate reference was replaced by updated reference.* *Only four studies, we think are very important to the topic and were published before 2010 (Hannah et al 2000, Hutton et al. 2003, Kainer et al. 2003, Grootscholten et al. 2008) were kept in the manuscript.*

A prospective analyses? is this trully prospective or retrospective? approval (Ref:176/18) means 2018?

*Thank you for pointing out this unclarity. Our study is an interim analysis of an ongoing larger single center study at our department starting from 2004. Our study design is overall prospective, but additional data was collected in a retrospective approach. The additional retrospective data collection was approved by the local ethics committee in 2018. An explanation was added in the revised manuscript.*

extremely low success rate of ECV -22% should be discussed and explained

*Thank you for addressing this important topic. In the rewritten discussion, we clarified the background of our low success rate.*

reults chapter is written badly - please rewrite.

*Thank you for your remarks, the results chapter has been rewritten. We focused on improving the language and cut out the repetitive results. I hope your demands were met.*

tables - ages with 2 decimals? what age does 33.13 represents?? parity and gravidity with decimals?

*The differences between the two groups in cases of age, parity, gravidity etc. were only visible within the decimals despite being significant. We shortened the decimal digits to only one digit, so the difference is still visible. Without the digits the numbers would be the same, although there is a significant difference.*

table 2 looks like it was copied from the SPSS sowtware - please provide a better version of presentation of table

*Thank you for the remark. The regression was run using the program Bias. We diminished the reported variables to Odds ratio, standard deviation and Wald’s p.*

what does constant mean?

*The constant term in regression analysis is the value at which the regression line crosses the y-axis, also known as the y-intercept. It was deleted from the table, as it may cause incomprehension.*

vaginal delivery rate of 62% is very low - should be discussed

*Thank you for pointing out this unclarity. The vaginal delivery rate of 62% refers to overall vaginal delivery after ECV disregarding the success. To make it clearer, we changed the labeling of the table to: ‘all deliveries after ECV’.*

*This number describes cephalic as well as breech vaginal delivery. The rate of cephalic vaginal delivery after successful ECV is 78%, which is higher than the nationwide average on cephalic birth. The rate of vaginal breech birth after unsuccessful ECV exceeds with 60% the nationwide average as well as rates described in previously published data.*

line 275-276 claiming novely - what about 31312960?

*Thank you for your remark. While Levin et al. offers ECV as an alternative between CS and breech delivery, at our center we offer a combined approach discussing ECV, vaginal breech delivery and CS to reach a shared decision with the patient.*

overall i dont see novelty, maybe unfamiliarity with current literature, therefore a more thorough literature review should be performed in the introduction and discussion and old references (more than 10 years should be discuraged)

*The reference list was updated, the introduction and discussion were rewritten. We hope your demands were met in the revised manuscript.*

limited new information, low success rate - thus limiting generalizability

*We addressed this point more precise in the revised discussion.*

Reviewer #2: First of all, this is a very interesting manuscript about management of breech presentation at term. This Care Unit seems to be a very expertise Breech Unit, and provide care about counseling about all possible maneuvers such as ECV, cesarean and vaginal birth. Congratulations for that.

However, it is commented on several occasions that this is the first article published about the safety of ECV in 38 weeks of gestation (lines 39, 49, 85, 275). The study fails to address how the findings relate to previous research in this area. The authors should return to publications such as that of Rodgers et al., 2017 (reference number 32 of the manuscript), where the mean gestational age of the ECV was 37 + 5, or in others such as Beuckens et al., 2015 (an observational study of the success and complications of 2546 external cephalic versions in low-risk pregnant women performed by trained midwifes) where 60% were more than 36 weeks; or in other publications like Ainsworth et al. 2017, with ECV even in 40 weeks of gestation. All of these publications are about ECV complications.

*Thank you for your remarks, we followed your recommendation and discussed the articles mentioned above alike your suggestions.*

Also, the complication total rate is 17%. This is higher than 6% in the revision published by Kok et al (2008) or Rodgers et al. (2017) about 5% or 2,5% in Neatherlands by Beuckens et al. (2016).

*Thank you for addressing this important issue. Our “higher” complication rate compared to previously published data is the result of reporting on all minor bradycardia and CTG-alterations. Beuckens et al. only reported on bradycardia lasting over 10 minutes, Kok et al. recommended only to report on bradycardia leading to CS. We discussed the complication rate in the revised paper.*

This manuscript can be very interesting and important, but the authors should rewrite their Introduction and Discussion to reference the related literature, because this is not the first publishing data about 38 gw ECVs.

*Thank you for your remarks. We rewrote the Introduction and Discussion as recommended and revised the reference list with more present studies.*
