# Supplementary material for: External cephalic version at 38 weeks’ gestation at a specialized German single center
Source: PLoS One. 2021 Aug 30;16(8):e0252702. doi: 10.1371/journal.pone.0252702 (PMC8405029; doi:10.1371/journal.pone.0252702)
Supplement: Attachment — Submitted filename: Response to Reviewers.docx [file pone.0252702.s002.docx]

**Rebuttal Letter PONE-D-20-24091R1**

Dear Editor, dear Reviewers,

thank you for your helpful and supporting input and questions you raised in this review process. Addressing each point improved the manuscript. Hopefully, your demands were met within our submitted revision.

Below, you can find a point-to-point statement. (Answers in *italic*)

Yours sincerely,

Ann-Sophie Zielbauer

1. Review Comments to the Author

Reviewer #3:

*Thank you for your profound comments. They were very helpful in improving our manuscript.*

38. Not clear what is meant by breech birth procedures?

*Thanks for the remark, the corresponding part has been specified and alternatives for breech birth were described.*

39. There has been a paper published this year that demonstrated that ECV at 38 weeks has equal success rates to ECVs performed at 36, 37, 39 and 40 weeks and is equally safe.

*Thank you for pointing out this recent paper. The paper was cited and addressed in the introduction and discussion of the revised manuscript.*

48. ECV at 38 weeks.

*Thank you very much for your remarks regarding grammar and spelling. We undertook a profound correction. Additionally, the manuscript was proof-read by the suggested English language editing service.*

49. False claim of first evidence.

*Thanks for this comment. This statement had been adjusted to the current evidence in the revised manuscript.*

131. No mention on the technique of ECV used, i.e., forward or backward flip?

*Again, thank you for the helpful remark. The explanation below was added in the revised manuscript.*

*“The baby was moved upwards with one hand and pushed to perform a forward or backward roll, preferably in the direction with less resistance. In cases of unsuccessful ECV (NECV), both directions were attempted. The direction (backwards or forwards) of successful ECV (SECV) has not been documented.*

154. fetuses.

*Thanks, we corrected the typo.*

160. Success rates appear to be skewed by the higher numbers of nulliparous women.

*Thank you very much for pointing out this important issue. The range of nulliparous women in our population is indeed higher compared to previously published data. We discussed the lower range in the revised manuscript.*

177. Definition of AFI measurements not mentioned, i.e., abundant is 20, 25, 30 cm?

*As suggested, we explained the cutoff for an abundant AFI score at our center.*

289. The study published in 2021 reported higher success with higher EFW.

*Thank you for the recommendation. We discussed this recent evidence in our submitted revision.*

290-293. Need to be re-written in a clearer way

*This part has been extended for more clarity. The whole manuscript has been double-checked for infelicitous wording.*

318. A fetus cannot be referred to as children.

*Thank you for pointing this out. We corrected this context.*

327 – 329. No justification/hypothesis written on to why success was higher in larger fetuses.

*Thanks again for the helpful comment. We discussed possible hypothesis for the observed higher success rate in larger fetuses.*

Reviewer #4:

I read with great interest the Manuscript titled “External cephalic version in 38 weeks’ gestation in a specialized German single center ”
The topic of this manuscript falls within the scope of PLOS One.


I was particularly pleased to review this revised version of the manuscript. In my honest opinion, the past reviewers' concerns have been resolved by the Authors. Now its scientific soundness is interesting enough to attract the readers’ attention. The methodology is accurate, and conclusions are supported by the summarized evidence.

However, I believe that the Discussions might benefit from a brief deeping of factors that might or might not improve the ECV successfulness, for example:
- Neuraxial analgesia [ PMID 27131581]
- Tocolysis [PMID 33421816]
- Other prognostic factors [PMID 31369397]

*Thank you very much for your profound comments and remarks. The suggested studies are very interesting and highly relevant. We discussed these articles alike your suggestions. We hope your demands were met in the revised manuscript. The language within the manuscript was also improved with the help of an editing service.*
